# A Triterpenoid‐Enriched GLE70 Fraction From *Ganoderma lingzhi* Ameliorates Alcoholic Liver Disease via Multi‐Target Regulation

**DOI:** 10.1002/fsn3.71893

**Published:** 2026-05-14

**Authors:** Yanjun Chen, Wenjia Li, Shiyi Ye, Jiaxue Li, Xiaobing Song, Qianli Ma, Ping Chen, Biying Zhang

**Affiliations:** ^1^ College of Food Science and Engineering Jilin Agricultural University Changchun Jilin Province China; ^2^ Zhongke Special Food Institute Changchun Jilin Province China

**Keywords:** alcoholic liver disease, antioxidant activity, *Ganoderma lingzhi* triterpenoids, network pharmacology and molecular docking

## Abstract

Alcoholic liver disease (ALD) arises from chronic alcohol consumption and progresses toward severe hepatic injury. *Ganoderma lingzhi* (*G. lingzhi*) triterpenoids possess notable hepatoprotective potential, yet the dense structure and complex chemical composition of the fruiting body make it challenging to obtain representative and bioactive fractions. In this study, an ultrasound‐assisted enzymatic extraction was used to prepare a *Ganoderma lingzhi* triterpenoid‐enriched extract (GLE), and its hepatoprotective effects were evaluated through physicochemical characterization, network pharmacology, and animal experiments. The optimized extraction yielded 16.32 ± 0.22 mg of total triterpenoids per gram of dry material. Functional screening identified GLE70 as the most active fraction, showing strong antioxidant activity, alcohol dehydrogenase activation, inhibition of carbohydrate‐hydrolysing enzymes, and bile acid binding capacity. HPLC‐TOF‐MS/MS analysis showed that GLE70 contained multiple triterpenoids, with eight relatively abundant components including ganoderic acids B and D. Network pharmacology and molecular docking predicted *AKT1*, *TNF*, and *SRC* as key targets associated with the hepatoprotective effects of GLE70. In vivo, GLE70 increased AKT1 expression, reduced TNF‐α and SRC‐associated inflammatory signals, and improved hepatic function, antioxidant status, and lipid metabolism, suggesting a coordinated multi‐target regulatory effect. Overall, this study obtained a bioactive triterpenoid‐enriched fraction from *G. lingzhi* and provided insights into its multi‐target regulatory characteristics in alleviating ALD, providing a basis for further application of *G. lingzhi* triterpenoids in liver protection.

## Graphical abstract

GLE fractions were prepared from *Ganoderma lingzhi* using gradient ethanol elution and evaluated for their biological activities. Among the fractions, GLE70 exhibited the strongest antioxidant and hepatoprotective effects. Integrated chemical characterization, network pharmacology, molecular docking, and in vivo validation suggested that GLE70 may exert protective effects against alcoholic liver disease through multi‐target regulation.

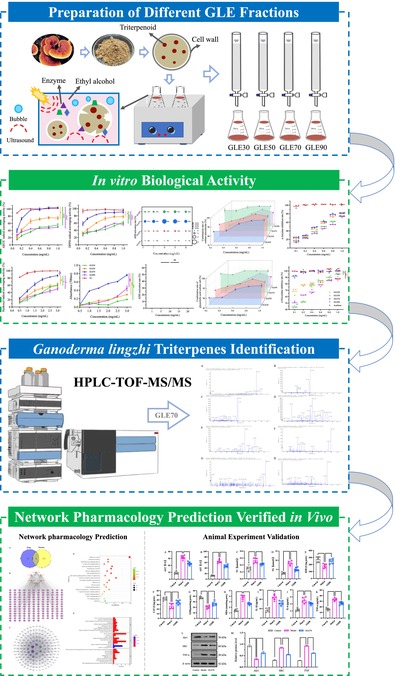

## 1 Introduction

Alcoholic liver disease (ALD), induced by chronic alcohol consumption, leads to progressive hepatic injury and imposes substantial health and socioeconomic burdens. Globally, 43% of the population consumes alcohol and more than 75 million individuals are diagnosed with alcohol‐related disorders, contributing to a 260‐fold increase in ALD‐associated mortality risk (Devarbhavi et al. 2023). Clinically, ALD progresses from alcohol‐induced hepatocellular injury to alcoholic fatty liver, hepatitis, cirrhosis, and ultimately hepatocellular carcinoma, driven by oxidative stress, inflammation, and metabolic dysfunction (Seitz et al. 2018; Wei et al. 2024). Current preventive and therapeutic strategies, including alcohol abstinence, lifestyle modification, and pharmacological intervention, remain inadequate, with limited therapeutic efficacy and notable adverse effects (Singal et al. 2018). This therapeutic gap underscores the urgent need for effective and safe adjunctive treatment strategies. Recently, medicinal and edible homologous substances, such as *G. lingzhi* (Zhao et al. 2025), *Dendrobium officinale* (Wang, Xiong, et al. 2025), *Astragalus membranaceus* (Lu et al. 2025), *Cistanche deserticola* (Wang, Wang, et al. 2025), and *Cornus officinalis* (Brodyak et al. 2025), have garnered growing research attention due to their multi‐target synergistic actions, low toxicity, and broad availability. Unlike single‐component pharmaceuticals, these substances exert coordinated effects through multiple bioactive constituents, thereby providing more comprehensive and stable therapeutic outcomes and offering a promising therapeutic paradigm for ALD intervention.

*G. lingzhi* is a highly valued medicinal and edible fungus with millennia of ethnopharmacological significance across Asia. To date, more than 400 compounds have been identified in *G. lingzhi*, including triterpenoids, polysaccharides, proteins, steroids, and nucleotides (Ahmad 2018). Among these constituents, triterpenoids represent an important class of metabolites in *G. lingzhi*, including compounds such as ganoderic acids, ganoderenols, and ganoderenes (Ryu et al. 2021). These triterpenoids exhibit a broad spectrum of biological activities, including antioxidant, antitumor, hypolipidemic, hypoglycemic, and anti‐HIV protease effects (Adeyi et al. 2021; Galappaththi et al. 2022; Guo et al. 2020). Increasing evidence indicates that *G. lingzhi* also exerts protective effects against ALD (Cao et al. 2022). Previous studies on individual triterpenoid constituents, such as ganoderic acid D, have shown that they can suppress ROS generation and modulate PERK/NRF2 signaling, thereby mitigating oxidative stress (Xu et al. 2020). However, these studies have mainly focused on isolated compounds, which may not fully capture the inherent multi‐component synergy and multi‐target characteristics of *G. lingzhi*. Increasing evidence suggests that whole extracts or multi‐component systems often exhibit enhanced biological effects compared to isolated constituents (Li and Vederas 2009; Zhao et al. 2020). Research has demonstrated that the dried whole‐plant *Artemisia annua* L. outperforms its isolated active component artemisinin in both reversing established malaria drug resistance and more importantly, markedly delaying the emergence of new resistance (Elfawal et al. 2015). This principle of multi‐component synergy extends to the hepatoprotective actions of *G. lingzhi*, where enhanced efficacy has been attributed to coordinated multi‐pathway regulation, including inhibition of lipid peroxidation, enhancement of antioxidant enzyme activity, and modulation of immuno‐inflammatory responses (Zhao et al. 2019).

Although *G. lingzhi* exhibits marked therapeutic potential against ALD, its compact and highly lignified fruiting‐body matrix hinders solvent penetration, making conventional extraction approaches such as organic solvent extraction time‐consuming, energy‐intensive, and limited in efficiency (Sharif Swallah et al. 2023). Moreover, such methods increase the likelihood of degrading thermolabile constituents, thereby compromising the integrity of key bioactive compounds and hindering industrial‐scale application. Accordingly, establishing a high‐yield, environmentally compatible, and extraction‐efficient process for recovering *G. lingzhi* triterpenoids is essential for fully exploiting its anti‐ALD potential. Ultrasound‐assisted enzymatic extraction integrates ultrasonic cavitation with enzymatic specificity, providing notable advantages in environmental sustainability and energy efficiency (Umego et al. 2021). In contrast to conventional thermal processes, this technique minimizes heat‐induced degradation during extraction and thereby preserves triterpenoid bioactivity (Shen et al. 2023). To overcome the pronounced interference from proteins and pigments typically present in crude GLE, an environmentally benign and low‐toxicity gradient ethanol purification system was established to selectively isolate bioactive fractions across different ethanol concentrations. This refinement is essential for accurately evaluating the bioactivity of *G. lingzhi* triterpenoids and for ensuring the reliability of downstream mechanistic investigations.

Although *G. lingzhi* triterpenoids exhibit promising hepatoprotective activity, the dense structure of the fruiting body makes extraction challenging, and most existing studies focus on individual triterpenoids or crude extracts, leaving the chemical composition of the enriched fractions and their functional relevance to ALD still to be fully elucidated. To address this issue, the present study employed an ultrasound‐assisted enzymatic extraction combined with AB‐8 macroporous resin–based fractionation to obtain representative triterpenoid‐enriched fractions in a function‐guided manner. These fractions were further examined through activity screening, chemical profiling, and target prediction, followed by network pharmacology, molecular docking, and an alcohol‐induced liver injury mouse model. This study aims to establish an analytical route that links enriched triterpenoid components with their potential molecular targets and biological relevance in ALD, thereby providing methodological support for clarifying the functional basis of *G. lingzhi* triterpenoids and advancing their application in ALD intervention.

## 2 Experimental Section

### 2.1 Materials

*Ganoderma lingzhi* was obtained from Jilin Mingxi Tang Ginseng Specialty Co. Ltd. and authenticated as *Ganoderma lingzhi* by DNA extraction, molecular sequencing, and sequence alignment. Neutral protease, hemicellulase, pectinase, cellulase, and papain were purchased from Solarbio (Beijing, China). AB‐8 macroporous resin and vanillin were obtained from Shanghai Yuanye Bio‐Technology Co. Ltd. (Shanghai, China). Oleanolic acid (≥ 98%, used as the standard for total triterpenoid determination) was purchased from Macklin (Shanghai, China). All solvents (HPLC grade) were supplied by Thermo Fisher Scientific.

### 2.2 Preparation of Crude GLE Extract

Powdered *G. lingzhi* (2 g) was used in each experiment and suspended in ethanol (1:20, w/v), adjusted to pH 5.0, and subjected to combined enzymatic hydrolysis and ultrasonication (100 W, 40 kHz). After enzyme inactivation at 90°C for 10 min, the extract was cooled and filtered, followed by vacuum concentration at 50°C and freeze‐drying for 10 h to obtain crude GLE. Five enzymes (pectinase, cellulase, neutral protease, hemicellulase, and papain) were initially screened at concentrations of 0.5%–3.0% under fixed extraction conditions to identify suitable candidates. Single‐factor experiments, including pH (4.5–6.5), extraction temperature (40°C–60°C), extraction time (40–120 min), and ethanol concentration (55%–95%), were then conducted to evaluate the effects of key process variables. Based on these results, a Box–Behnken design was employed for further optimization, in which the variables (X_1_–X_4_) were tested at three levels (−1, 0, +1) to determine the optimal extraction conditions. Samples for subsequent experiments were prepared at a larger scale under the optimized conditions.

### 2.3 Separation of GLE Using Macroporous Resin

GLE was loaded onto an AB‐8 macroporous resin column (3 × 60 cm; bed volume ~400 mL). One gram of GLE was dissolved in 15 mL anhydrous ethanol and applied to the column. Gradient elution was carried out with 30%, 50%, 70%, and 90% ethanol at 2 mL/min for two bed volumes. Each fraction was collected, concentrated under reduced pressure at 50°C, and freeze‐dried to obtain GLE30, GLE50, GLE70, and GLE90.

### 2.4 Determining the Total Triterpenoid Yield From *G. lingzhi*

Total triterpenoid equivalents were quantified using the vanillin–perchloric acid colorimetric method (R. Deng et al. 2025). Briefly, a 0.2 mL sample was reacted with 0.2 mL 5% vanillin–acetic acid and 0.8 mL perchloric acid at 70°C for 15 min, cooled, mixed with 4 mL ethyl acetate, and measured at 546 nm. Triterpenoid content and total yield (ω, mg/g) were calculated using the standard curve Y=9.715x−0.0141 (*R* ^2^ = 0.9981) and Formulas (1) and (2), where M_0_ is the triterpenoid mass (mg) from absorbance (A) via slope (a) and intercept (b), and M is the initial *G. lingzhi* powder mass (g).

(1) $$M_{0}=\left(A+b\right)/a$$

(2) $$\omega =\left(M_{0}\times \text{dilution factor}\right)/M$$

### 2.5 In Vitro Functional Activity Assays

#### 2.5.1 Antioxidant Capacity Assays

##### 2.5.1.1 DPPH Scavenging Activity Assay

Sample solutions (0.1–1.0 mg/mL) were mixed with an equal volume of 0.1 mmol/L DPPH and incubated in the dark at room temperature for 30 min. Absorbance was recorded at 517 nm, with ascorbic acid serving as the positive control (Zhang et al. 2023). The scavenging rate (%) was calculated using Equation (3), where A_sample,_ A_control_, and A_0_ denote the absorbance of the sample, control, and blank, respectively.

(3) $$\begin{array}{c} \text{Scavenging rate}\;\left(\%\right)\;\text{of DPPH},{\text{ABTS}}^{+},\\ \text{and}\cdot \mathrm{OH}=1-\left(A_{\text{sample}}-A_{\text{control}}\right)/A_{0} \end{array}$$

##### 2.5.1.2 ABTS ^+^ Scavenging Activity Assay

Samples (0.1–1.0 mg/mL) were mixed with ABTS^+^ solution at a 1:1 ratio and incubated in the dark for 10 min. Absorbance was measured at 734 nm, with ascorbic acid as the positive control (R. Zhang et al. 2023). The scavenging rate was calculated using equation (3).

##### 2.5.1.3 Hydroxyl Radical Scavenging Activity Assay

To 50 μL of sample (0.5–3.0 mg/mL), 50 μL each of salicylic acid–ethanol solution (9 mmol/L), FeSO_4_ (9 mmol/L), and H_2_O_2_ (0.3%) were added. The mixture was incubated at 37°C for 30 min, and absorbance was measured at 510 nm (Xu et al. 2019). Ascorbic acid was used as the positive control, and the ·OH scavenging rate was calculated using equation (3).

##### 2.5.1.4 Total Reducing Power Assay

Samples (0.2–1.0 mg/mL) were mixed with phosphate buffer (pH 6.6) and 1% K_3_[Fe(CN)_6_], incubated at 50°C for 20 min, reacted with 10% trichloroacetic acid, and the mixture was centrifuged at 3000 *g* for 10 min. The supernatant (2.5 mL) was combined with 0.5 mL of 0.1% FeCl_3_ and allowed to stand for 10 min (Xu et al. 2019). Absorbance was measured at 700 nm.

#### 2.5.2 Alcohol Dehydrogenase Activity Assay

Samples (1.0–20.0 mg/mL, 5 μL) were mixed with 75 μL of Na_4_P_2_O_7_ buffer (pH 8.8), 50 μL of 0.027 mol/L NAD^+^, and 25 μL of 11.5% ethanol. The plate was incubated at 37°C for 5 min, after which 5 μL of ADH solution (0.15 U/mL) was added. Absorbance at 340 nm (A_340_) was recorded every 10 s for 5 min (Vallee and Hoch 1955). ADH activity was calculated using Equation (4), where ΔA_340_/min (AU/min) represents the change in absorbance at 340 nm per minute. In Equation (5), A₁ denotes the ADH activity measured in the presence of the sample, while A_0_ represents the activity of the blank control.

(4) $$\begin{array}{c} \mathrm{ADH}\;\text{activity}\;\left(U/\mathrm{mL}\right)=\left(\Delta A_{340/\min}\times \text{total reaction volume}\times 10^{6}\right)/\\ \left(\text{enzyme content}\times 6.2\times 10^{3}\right) \end{array}$$

(5) $$\mathrm{ADH}\;\text{activation rate}\;\left(\%\right)=\left(A1-A0\right)/A0\times 100\%$$

#### 2.5.3 In Vitro Bile Acid‐Binding Assay

Sample solutions (1.0–20.0 mg/mL, 1 mL) were mixed with 1 mL pepsin (10 mg/mL) and 3 mL 0.01 M HCl, incubated at 37°C for 1 h (pH 1.5), then adjusted to pH 6.3. Subsequently, 4 mL trypsin (10 mg/mL) was added for 1 h, followed by 4 mL 1 mM bile salt for another hour. After centrifugation (3500 g, 20 min), the supernatant was measured at 387 nm (Chang et al. 2024), and the bile salt binding rate was calculated using equation (6), where C₁ and C_2_ represent the initial and final bile salt concentrations (μmol/L), respectively.

(6) $$\text{Bile acid salt binding rate}\;\left(\%\right)=\left(\;C1-C2\;\right)/C1\times 100\%$$

#### 2.5.4 In Vitro Carbohydrate‐Digesting Enzyme Inhibition Assay

##### 2.5.4.1 α‐Amylase Inhibitory Activity Assay

Samples (30 μL, 0.1–1.0 mg/mL) were mixed with 30 μL α‐amylase (10 U/mL) and incubated at 37°C for 15 min. After adding 30 μL of 1% soluble starch and incubating for another 15 min, 50 μL DNS reagent was added and the mixture was boiled for 10 min. Following ice cooling and volume adjustment to 1 mL, absorbance was measured at 540 nm (Nsor‐Atindana et al. 2020). Inhibition was calculated using Formula (7), with acarbose as the positive control, where A_0_, A_1_, A_2_, and A_3_ represent blank, sample reaction, enzyme‐replaced, and sample/enzyme‐replaced absorbances, respectively.

(7) $$\alpha ‐\text{amylase or}\;\alpha ‐\text{glucosidase inhibition rate}\;\left(\%\right)=1-\left(A_{1}-A_{2}\right)/\left(A_{0}-A_{3}\right)\times 100\%$$

##### 2.5.4.2 α‐Glucosidase Inhibitory Activity Assay

Samples (40 μL, 0.1–1.0 mg/mL) were mixed with 20 μL α‐glucosidase (100 U/mL) and 40 μL phosphate buffer (pH 6.8), incubated at 37°C for 15 min, then 40 μL PNPG (10 mmol/L) was added for another 15 min. The reaction was stopped with 80 μL Na_2_CO_3_ (0.2 M), and absorbance was measured at 405 nm (Ryu et al. 2021). Inhibition was calculated using formula (7).

### 2.6 HPLC‐TOF MS/MS Analysis of GLE70

#### 2.6.1 HPLC‐TOF‐MS/MS Analysis in Positive Ion Mode

Samples were analyzed on a Waters ACQUITY UPLC BEH C8 column (1.7 μm, 2.1 × 100 mm) at 50°C with a 4 μL injection volume. The mobile phases were 0.1% formic acid in water (A) and acetonitrile (B) at 0.3 mL/min. The gradient was: 0–2 min, 5% B; 2–37 min, 5%–99% B; 37–42 min, 99% B; 42.1–45 min, 5% B. MS acquisition used full MS and DDA MS/MS with source temperature 550°C, curtain gas 35 psi, declustering potential 80 V, collision energy 10 eV, MS/MS energy 40 ± 15 eV, MS^1^ scan 70–1050 Da, and MS^2^ scan 50–1050 Da.

#### 2.6.2 HPLC‐TOF‐MS/MS Analysis in Negative Ion Mode

Negative ion mode used a Waters ACQUITY UPLC HSS T3 column (1.8 μm, 2.1 × 100 mm) at 50°C with a 4 μL injection volume. Mobile phase A was 6.5 mM ammonium bicarbonate in water, and B was 6.5 mM ammonium bicarbonate in 90% methanol/water, at 0.3 mL/min. The gradient was: 0–2 min, 5% B; 2–42 min, 5%–99% B; 42–47 min, 99% B; 47.1–50 min, 5% B. MS conditions were: source temperature 350°C, curtain gas 35 psi, declustering potential −80 V, collision energy −10 eV, MS/MS energy −35 ± 15 eV, MS^1^ scan 70–1050 Da, and MS^2^ scan 50–1050 Da.

### 2.7 Network Pharmacology

SMILES for *G. lingzhi* triterpenoids were retrieved from PubChem and submitted to SwissTargetPrediction to predict targets. ALD‐related targets were obtained from GeneCards, TTD, and OMIM; after deduplication, compound–disease overlaps were identified (Venny) and visualized. A compound–target–disease network and PPI (STRING, confidence ≥ 0.4) were built and analyzed in Cytoscape 3.9.1 to identify core nodes, followed by GO and KEGG enrichment using DAVID, with results plotted via standard bioinformatics tools.

### 2.8 Molecular Docking of Triterpenoids With Target Proteins

2D compound structures were obtained from UniProt and converted to energy‐minimized 3D conformations using Chem3D 19.0. Human target protein structures were retrieved from UniProt in PDB format. Molecular docking was performed in AutoDockTools 1.5.7, and results were visualized with PyMol 3.2.

### 2.9 In Vivo Validation Experiment

#### 2.9.1 Animal Treatment and Sample Collection

Six‐week‐old SPF‐grade male C57BL/6 mice (20 ± 2 g) were obtained from the Experimental Animal Center of Jilin Agricultural University (License No. SCXK [Beijing] 2019‐0010; Ethics Approval No. 20240930002) and acclimated for 1 week under a 12 h light/dark cycle at 22°C ± 2°C and 50% ± 20% relative humidity. Mice were randomly assigned to Control, Model, or GLE70 groups (*n* = 6). For 14 days, all had ad libitum access to food and water. Gavage was performed twice daily: Control, saline; Model, 52% liquor (10 mL/kg) morning and saline evening; GLE70, liquor morning and GLE70 (100 mg/kg) evening. After the final gavage, mice were fasted 12 h (water allowed), anesthetized with pentobarbital, and blood and liver samples were collected. Serum was obtained by centrifugation (3500 rpm, 10 min, 4°C), and liver tissues (0.1 g) were homogenized in nine volumes of ice‐cold saline and centrifuged under the same conditions.

#### 2.9.2 Biochemical Analysis of Serum and Hepatic Samples

Serum aspartate aminotransferase (AST), alanine aminotransferase (ALT), total cholesterol (TC), and triglyceride (TG) were measured using Jiancheng Bioengineering kits (Nanjing, China). Liver supernatants were used for protein quantification and to assess superoxide dismutase (SOD), glutathione (GSH), catalase (CAT) activities, malondialdehyde (MDA) levels, and tumor necrosis factor‐alpha (TNF‐α), interleukin‐1 beta (IL‐1β), and interleukin‐6 (IL‐6) concentrations by ELISA (Shanghai Youxuan, China) following the manufacturers' protocols.

#### 2.9.3 Western Blot Analysis of AKT1, SRC and TNF‐α Proteins

Liver tissue (20 mg) was homogenized in RIPA buffer with 1% PMSF, and protein concentration was determined by BCA assay. Proteins (10 μL) with Marker (5 μL) were separated by SDS‐PAGE (60 V until entering the gel, then 120 V), transferred to methanol‐activated PVDF membranes, blocked, incubated overnight at 4°C with primary antibodies against AKT1, SRC, and TNF‐α, followed by secondary antibodies for 1 h, and bands were visualized by chemiluminescence and quantified using ImageJ.

### 2.10 Statistical Analysis

All measurements were performed at least in triplicate. All data are presented as mean ± standard deviation (SD). Statistical analysis was performed using SPSS 20.0. Differences among groups were analyzed by one‐way ANOVA followed by Duncan's multiple range test. *p < 0.05 and **p < 0.01 were considered statistically significant.

## 3 Results and Discussion

### 3.1 Ultrasound‐Assisted Enzymatic Extraction of GLE

#### 3.1.1 Enzyme Screening Effects on Triterpenoid Yield

**FIGURE 1:**
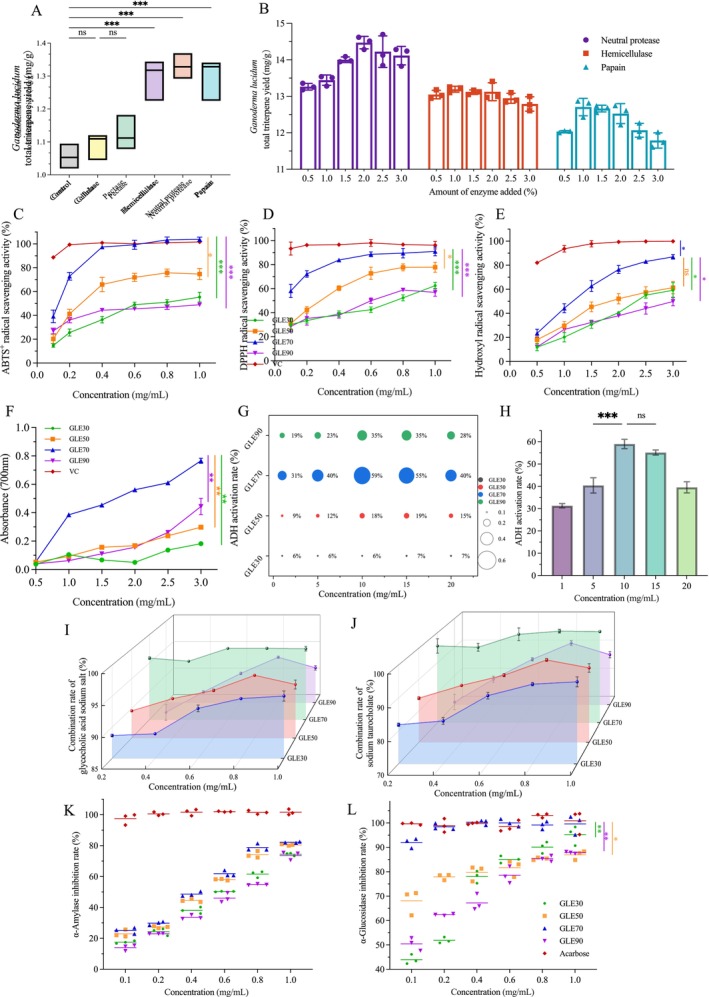
Effects of enzyme type (A) and enzyme concentration (B) on the triterpenoid yield of *G. lingzhi*. Antioxidant activities of GLE fractions. DPPH (C), ABTS^+^ (D), hydroxyl radical scavenging (E), and reducing power (F). Biological functions of GLE fractions. ADH activation (G), dose–response of GLE70 (H), binding to glycocholate (I) and taurocholate (J), and inhibition of α‐amylase (K) and α‐glucosidase (L). Statistical significance: *p < 0.05, **p < 0.01, ***p < 0.001. Unless otherwise indicated, asterisks denote comparisons with the positive control (ascorbic acid for panels C–H and acarbose for panels K–L). For panels I–J, asterisks denote comparisons with the GLE70 group.

To improve the release of triterpenoids from the rigid *G. lingzhi* matrix, five enzymes were initially evaluated. As shown in Figure 1A, hemicellulase, neutral protease, and papain exhibited the most pronounced enhancement in triterpenoid yield and were therefore selected for subsequent optimization. Hemicellulase degrades plant cell‐wall polysaccharides (De Souza and Kawaguti 2021), facilitating the release of intracellular compounds, whereas papain and neutral protease hydrolyze structural proteins (Tacias‐Pascacio et al. 2021), further promoting cell‐wall disruption. As illustrated in Figure 1B, the effective concentration range was 1.5%–2.5% for neutral protease and 0.5%–1.5% for both hemicellulase and papain. Based on orthogonal analysis (Tables S1–S2), a combined enzyme system consisting of 1.5% neutral protease, 0.5% hemicellulase, and 1.0% papain produced a triterpenoid yield of 15.65 ± 0.26 mg/g. Variance analysis indicated no significant difference among the individual enzymes within the tested ranges, supporting the use of this composite enzyme system for subsequent extraction optimization.

#### 3.1.2 Response Surface Optimization of Triterpenoid Extraction

Single‐factor analyzes (Figure S1) identified the effective ranges of key parameters for ultrasound‐assisted enzymatic extraction, including pH 4.5–5.5, extraction temperature 45°C–55°C, extraction time 60–100 min, and ethanol concentration 75%–95%. Based on these results, a Box–Behnken response surface design was established (Tables S3–S4; Figure S2). The model predicted optimal conditions of pH 5.04, extraction temperature 52.31°C, extraction time 75.23 min, and ethanol concentration 87.08%, yielding a theoretical triterpenoid content of 16.35 mg/g. Considering practical operability, these parameters were adjusted slightly to pH 5.0, 52°C, 75 min, and 87% ethanol. Under these conditions, an average yield of 16.32 ± 0.22 mg/g was obtained, deviating from the theoretical value by only 0.003%, confirming the accuracy of the model. Compared with the ultrasound‐assisted enzymatic method of Do Dat et al., which reported a yield of 11.65 mg/g, the yield achieved in this study increased by 40.34% (Do Dat et al. 2022), demonstrating the efficiency of the optimized extraction process.

### 3.2 Analysis of Different GLE Fractions

Triterpenoid‐enriched extracts of *G. lingzhi* were prepared using an ultrasound‐assisted enzymatic method and separated on AB‐8 macroporous resin. Gradient elution with ethanol solutions of varying concentrations yielded four fractions, GLE30, GLE50, GLE70, and GLE90, with total triterpenoid contents of 164.2, 204.5, 354.6, and 242.0 mg/g, respectively. Among them, GLE70 exhibited the highest content, indicating that triterpenoids were enriched in the 70% ethanol fraction. These results suggest that the 70% ethanol fraction may be the most suitable for obtaining triterpenoid‐rich components and was therefore selected for subsequent analyzes.

### 3.3 In Vitro Activity Assays of Different GLE Fractions

#### 3.3.1 Antioxidant Activity of Different GLE Fractions In Vitro

Oxidative stress is a central driver of ALD progression (Asrani et al. 2019), and the antioxidant properties of each extract were therefore evaluated. As shown in Figure 1C, all fractions exhibited dose‐dependent DPPH scavenging activity within the tested range. At 1.0 mg/mL, GLE70 showed scavenging rate (90.97% ± 3.69%) with an IC_50_ of 0.0693 mg/mL, significantly higher than GLE50 (*p* < 0.05) and comparable to the positive control (*p* > 0.05). A similar trend was observed for ABTS^+^ scavenging (Figure 1D), GLE70 demonstrated markedly stronger activity than GLE50 (*p* < 0.05), achieving a scavenging rate of 103.47% ± 2.40% at 0.8 mg/mL (IC_50_ = 0.1231 mg/mL), again comparable to the positive control (*p* > 0.05). For hydroxyl radical scavenging (Figure 1E), GLE70 outperformed GLE30 and GLE90 (*p* < 0.05), reaching 86.97% ± 1.88% at 3.0 mg/mL (IC_50_ = 1.073 mg/mL). Consistently, GLE70 also exhibited the greatest reducing power among all fractions, with an absorbance of 0.77 ± 0.02 at 3.0 mg/mL (Figure 1F), which was significantly higher than that of the other fractions (*p* < 0.01). Previous studies have shown that GLE possesses dose‐dependent free‐radical scavenging properties (Adeyi et al. 2021; Saltarelli et al. 2019). The superior antioxidant activity of GLE70 observed here is likely attributable to the synergistic interactions among multiple triterpenoids enriched in this fraction. Overall, GLE70 displayed the most potent antioxidant capacity across all assays, indicating its strong potential in mitigating oxidative stress–related hepatic injury.

#### 3.3.2 ADH Activation Rate of Different GLE Fractions In Vitro

ADH is a cytosolic zinc‐dependent oxidoreductase that uses NAD^+^ as an essential cofactor to catalyze the first step of hepatic ethanol metabolism. Nearly 90% of ingested ethanol is oxidized through this pathway, in which ethanol is converted to acetaldehyde by ADH and subsequently to acetic acid by aldehyde dehydrogenase (Deng et al. 2014; Jiang et al. 2020; Xiao et al. 2018). Given the central role of ADH in determining ethanol clearance efficiency, the activation of this enzyme by different GLE fractions was evaluated. Figure 1G indicates that the ADH activation profiles of GLE50, GLE70, and GLE90 followed a characteristic pattern: activity rose with increasing concentrations and declined at higher levels, likely due to enzyme saturation or increased solution viscosity affecting catalytic dynamics. Among all fractions, GLE70 showed a superior activation capacity compared with the other fractions, reaching a maximum activation rate of 58.99% ± 2.08% at 10 mg/mL (Figure 1H), which was significantly higher than that at 5 mg/mL (*p* < 0.01). When benchmarked against reported natural extracts, GLE70 demonstrated remarkable potency; its maximal ADH activation exceeded that of lotus root methanol extract and bay leaf aqueous extract by 3.13‐fold and 1.42‐fold, respectively (Jung et al. 2023; Yang et al. 2023). These data suggest that GLE70 markedly enhances ethanol metabolism efficiency and may contribute to accelerated ethanol clearance in vivo.

#### 3.3.3 Bile Acid‐Binding Capacity of GLE Fractions

Cholesterol dysregulation and hepatic lipid deposition are early pathological features of ALD. In normal physiology, cholesterol is converted into bile acids, which subsequently bind to glycine or taurine to form bile salts; these complexes are excreted or reabsorbed through enterohepatic circulation, thereby modulating lipid homeostasis (Yang et al. 2024). Therefore, the bile salt–binding capacity of GLE fractions provides indirect insight into their potential lipid‐lowering effect. The concentration–response curves depicted in Figure 1I,J demonstrate a clear ascending trend in bile salt‐binding efficiency across all fractions. GLE70 exhibited the highest activity, with binding rates of 97.55% ± 0.15% for glycocholic acid sodium and 100.48% ± 0.60% for taurocholic acid sodium at 0.8 mg/mL. Notably, this performance surpassed that of Pleurotus tuber‐regium sclerotium, which required 80 mg/mL to reach its maximum binding capacity (C. Wang et al. 2022), underscoring the significantly higher potency of GLE70. Given the abundant triterpenoids, including ganoderic acids G, B, H, A, and F, previously reported to contribute to lipid regulation (Tong et al. 2023), the superior bile acid‐binding ability of GLE70 may reflect synergistic interactions among multiple bioactive triterpenoids enriched in this fraction.

#### 3.3.4 Carbohydrate‐Digesting Enzyme Inhibitory Activity of GLE Fractions

Chronic alcohol consumption disrupts glucose metabolism partly by reducing circulating insulin‐like growth factor 1 and impairing insulin signaling, ultimately contributing to hepatic insulin resistance (Liu et al. 2024). Plant‐derived compounds can ameliorate these metabolic disturbances by inhibiting α‐amylase and α‐glucosidase, the major enzymes responsible for starch hydrolysis and intestinal glucose release (Shin et al. 2019). Therefore, evaluating the inhibitory effects of GLE fractions on these enzymes helps clarify their potential role in modulating glucose metabolism under ALD conditions. The inhibitory profiles of GLE fractions against α‐amylase are shown in Figure 1K,L. All fractions exhibited dose‐dependent inhibition, with maximal inhibition at 1.0 mg/mL ranging from 73.65% to 82.13%. GLE70 demonstrated one of the highest inhibitory activities, significantly differing from acarbose (*p* < 0.01). Similarly, GLE70 exhibited robust α‐glucosidase inhibition, reaching an IC_50_ of 0.03844 mg/mL, comparable to acarbose and significantly stronger than the other fractions (*p* < 0.05 or *p* < 0.01) (Chen et al. 2018; Guo et al. 2021). By suppressing both α‐amylase and α‐glucosidase, GLE70 may slow carbohydrate digestion, reduce postprandial glucose spikes, and lessen metabolic stress on hepatocytes (Oboh et al. 2021). Together with its potent antioxidant activity, strong ADH activation, and high bile acid‐binding capacity, these carbohydrate‐digesting enzyme inhibitory effects reinforce the multi‐functional nature of GLE70 and support its selection for further structural and in vivo investigations.

The in vitro results demonstrate that GLE70 exhibits multiple functional activities. Its antioxidant properties contribute to the alleviation of oxidative stress, while enhanced ADH activity indicates a role in promoting alcohol metabolism. Its bile acid‐binding capacity, together with the inhibitory effects on α‐amylase and α‐glucosidase, indicates its involvement in the regulation of glucose and lipid metabolism. These effects collectively target interconnected pathological processes in ALD, including oxidative damage, metabolic dysregulation, and inflammatory responses, which may contribute to its hepatoprotective potential.

### 3.4 HPLC‐TOF‐MS/MS Analysis of Triterpenoids in GLE70

Following HPLC–TOF–MS/MS analysis of GLE70, this study focused on the eight triterpenoids (the top 8 according to peak area percentage) with the highest relative abundances as the major constituents of the extract, as shown in Table 1. The identification was based on retention times, quasi‐molecular ions, MS/MS fragmentation patterns (Figure S3), and comparison with public mass spectrometry databases and previous literature. In negative‐ion mode, Ganoderic acid B showed the highest relative abundance, followed by Ganolucidic acid B. In positive‐ion mode, the major compounds ranked by peak area were Ganoderic acid D, Ganoderic acid LM2, Ganoderenic acid C, 9 (11)‐Dehydromanogenin, Ganodermic acid Jb, and a highly oxygenated lanostane‐type triterpenoid [4,4,8,10,14‐pentamethyl‐17‐(4,5,6‐trihydroxy‐6‐methylheptan‐2‐yl)‐2,5,6,7,9,15‐hexahydro‐1H‐cyclopenta[a]phenanthrene‐3,16‐dione] (Biswal et al. 2022; Guo et al. 2012, 2013; Nakagawa et al. 2018; Saltarelli et al. 2019).

**TABLE 1:** Identification results of triterpenoid components in GLE70.

Peak No.	Tentative identification	Rt (min)	Molecular formula	Ion forms	Precursor ion	Area	Fragmentation pattern (MS2, m/z)
1	Ganoderic acid B	15.29	C_30_H_44_O_7_	[M‐H]^−^	515.30007	1,779,571	497.2912 435.2915
2	Ganolucidic acid B	17.41	C_30_H_46_O_6_	[M + HCOO]^−^	547.32704	135,495	501.3211 483.3122
3	Ganoderic acid LM2	12.90	C_30_H_42_O_7_	[M + H]^+^	515.29997	739,124	497.2906 479.2801
4	Ganoderic acid D	14.45	C_30_H_42_O_7_	[M + H]^+^	515.29884	8,891,533	497.2888 451.2837
5	Ganoderenic acid C	15.76	C_30_H_44_O_7_	[M + H]^+^	517.31630	669,215	499.3083 481.2978
6	4,4,8,10,14‐Pentamethyl‐17‐ (4,5,6‐trihydroxy‐6‐methylheptan‐2‐yl)‐2,5,6,7,9,15‐hexahydro‐1H‐cyclopenta[a]phenanthrene‐3,16‐dione	16.19	C_30_H_46_O_5_	[M‐H_2_O + H]^+^	469.33213	10,096	469.3334 451.3275
7	9 (11)‐Dehydromanogenin	16.89	C_27_H_40_O_5_	[M + H]^+^	445.29545	83,431	445.2962 427.2845
8	Ganodermic acid Jb	19.30	C_30_H_46_O_4_	[M‐H_2_O + H]^+^	453.33722	10,668	453.3337 435.3297

### 3.5 Network Pharmacology and Molecular Docking

#### 3.5.1 Network Pharmacology Analysis of GLE70

**FIGURE 2:**
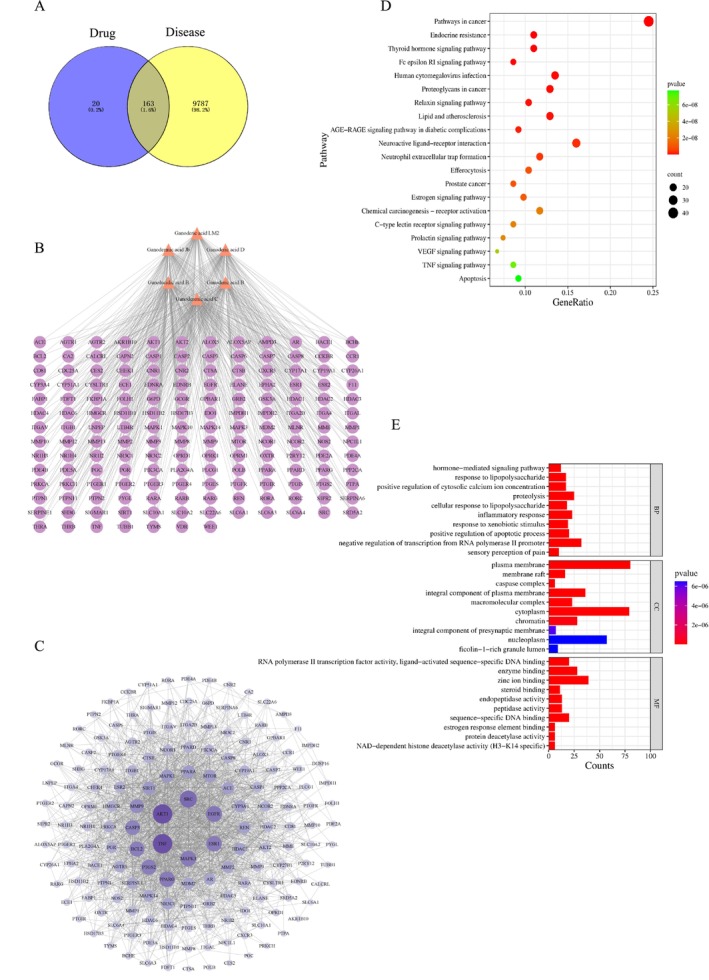
Network pharmacology analysis of GLE70 against ALD. (A) Venn diagram showing the overlap between predicted GLE70 targets and ALD‐related targets. (B) Component–target network, where orange triangles represent GLE70 triterpenoids and purple nodes denote intersecting targets. (C) PPI network illustrating connectivity density, with larger nodes indicating higher interaction degrees. (D) KEGG pathway enrichment, with circle size reflecting the number of genes associated with each pathway. (E) GO enrichment analysis summarizing major biological processes, cellular components, and molecular functions.

Based on the predicted targets of the identified GLE70 constituents, intersection with ALD‐related targets resulted in 163 shared genes (Figure 2A). The compound–target network (169 nodes, 549 edges; Figure 2B) and the PPI network (162 nodes, 1613 edges; Figure 2C) collectively identified *AKT1*, *TNF*, *SRC*, *EGFR*, and *MAPK3* as high‐degree hubs. AKT1 functions as a central regulator of hepatocellular survival and redox homeostasis (Shiau et al. 2022). TNF signaling governs proinflammatory amplification and hepatocyte injury through TNF‐α–dependent NF‐κB activation (Zhou et al. 2003). SRC serves as a critical upstream node coordinating PI3K–Akt, MAPK, and STAT3 pathways, thereby integrating inflammatory and metabolic stress responses (Jiang et al. 2022). These hubs show consistency with the observed in vitro bioactivities of GLE70, including marked antioxidant capacity, ADH activation, and bile acid–binding activity, suggesting convergent regulation of oxidative, inflammatory, and metabolic axes. KEGG analysis identified 151 enriched pathways, with prominent involvement of TNF signaling, PI3K–Akt signaling, lipid metabolic processes, proteoglycan‐associated pathways, neuroactive ligand–receptor interactions, and viral infection–related processes (Figure 2D). These pathways encompass major pathophysiological dimensions of ALD, including inflammation, oxidative injury, lipid dysregulation, and extracellular matrix remodeling. GO enrichment yielded 704 functional terms spanning biological processes, cellular components, and molecular functions (Figure 2E), principally related to oxidative stress modulation, inflammatory regulation, lipid metabolic processes, and hepatocellular defense mechanisms. The convergence of these functional categories suggests that GLE70 triterpenoids may exert hepatoprotective effects through a coordinated, multi‐target regulatory framework. In summary, the network pharmacology findings suggest that *G. lingzhi* triterpenoids may be involved in the modulation of pathways related to ALD progression of inflammatory signaling, cytoprotective redox pathways, and lipid metabolic homeostasis, consistent with the in vitro functional profile of GLE70.

#### 3.5.2 The Docking Simulation of Protein and *G. lingzhi* Triterpenoids

**FIGURE 3:**
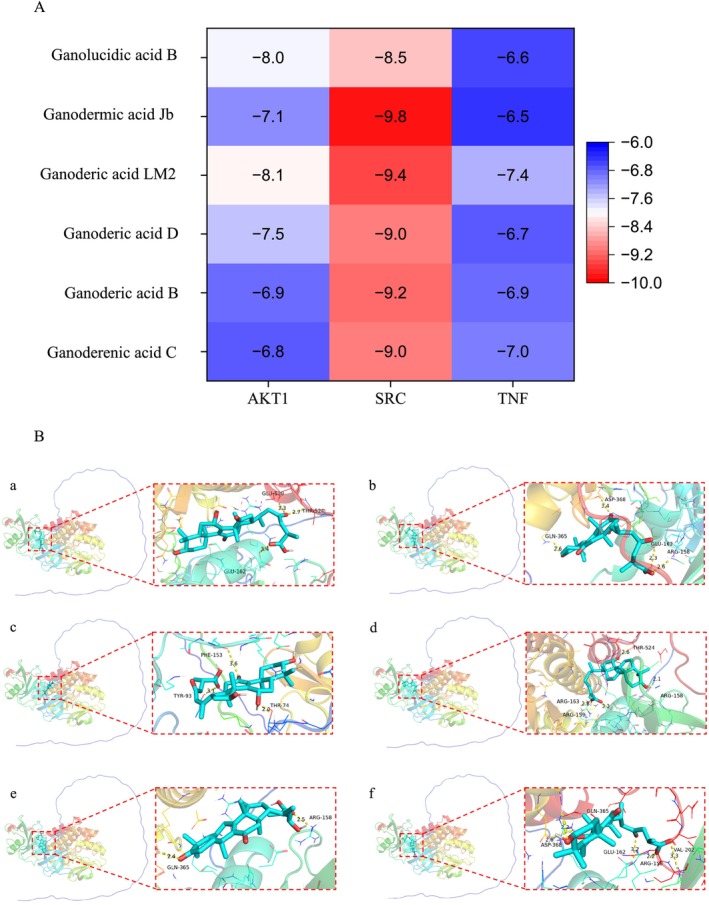
Heat map of binding energies between GLE70 triterpenoids and receptor proteins, and representative docking conformations of six major molecules with SRC including (A) Ganoderic acid B, (B) Ganoderic acid D, (C) Ganoderenic acid C, (D) Ganodermic acid Jb, (E) Ganoderic acid LM2, and (F) Ganolucidic acid B.

The triterpenoids in GLE70 (Ganoderic acids B, D, LM2, Ganoderenic acid C, Ganodermic acid Jb, and Ganolucidic acid B) were docked with the core ALD‐related targets *AKT1*, *TNF*, and *SRC* predicted by network pharmacology (Figure 3A). Binding energies below −5 kJ/mol indicated stable ligand–protein interactions (Alzarea et al. 2022). Among the targets, SRC displayed the strongest overall affinity for the triterpenoids, suggesting that these compounds may suppress SRC kinase activity, thereby attenuating hepatic stellate cell activation and inflammatory amplification in ALD. The observed interactions with AKT1 and TNF further support the potential of GLE70 constituents to engage multiple pathogenic processes simultaneously. Docking analysis revealed a conserved SRC binding pocket defined primarily by residues ARG‐158 and GLN‐365 (Figure 3B). Ganoderic acids D, Jb, B, and LM2 formed hydrogen bonds with ARG‐158 (2.1–3.3 Å) and GLN‐365 (2.4–3.4 Å), while Ganoderic acid C generated a short hydrogen bond with THR‐74 (2.0 Å), contributing additional structural stabilization. Ganoderic acid Jb exhibited multiple interactions involving ARG‐158, ARG‐159, and ARG‐163, consistent with its highest predicted binding affinity. Notably, the identified triterpenoid constituents in GLE70 exhibited consistent binding affinities toward the predicted core targets, thereby supporting a potential correspondence between the identified triterpenoids and their predicted molecular targets. Collectively, the docking results corroborate the predictions made by network pharmacology and highlight SRC, AKT1, and TNF as key molecular nodes through which GLE70 triterpenoids may exert hepatoprotective effects.

### 3.6 Experimental Validation of Anti‐ALD Effects

#### 3.6.1 Effects of GLE70 on Serum Liver Injury Markers and Lipid Profiles in ALD Mice

**FIGURE 4:**
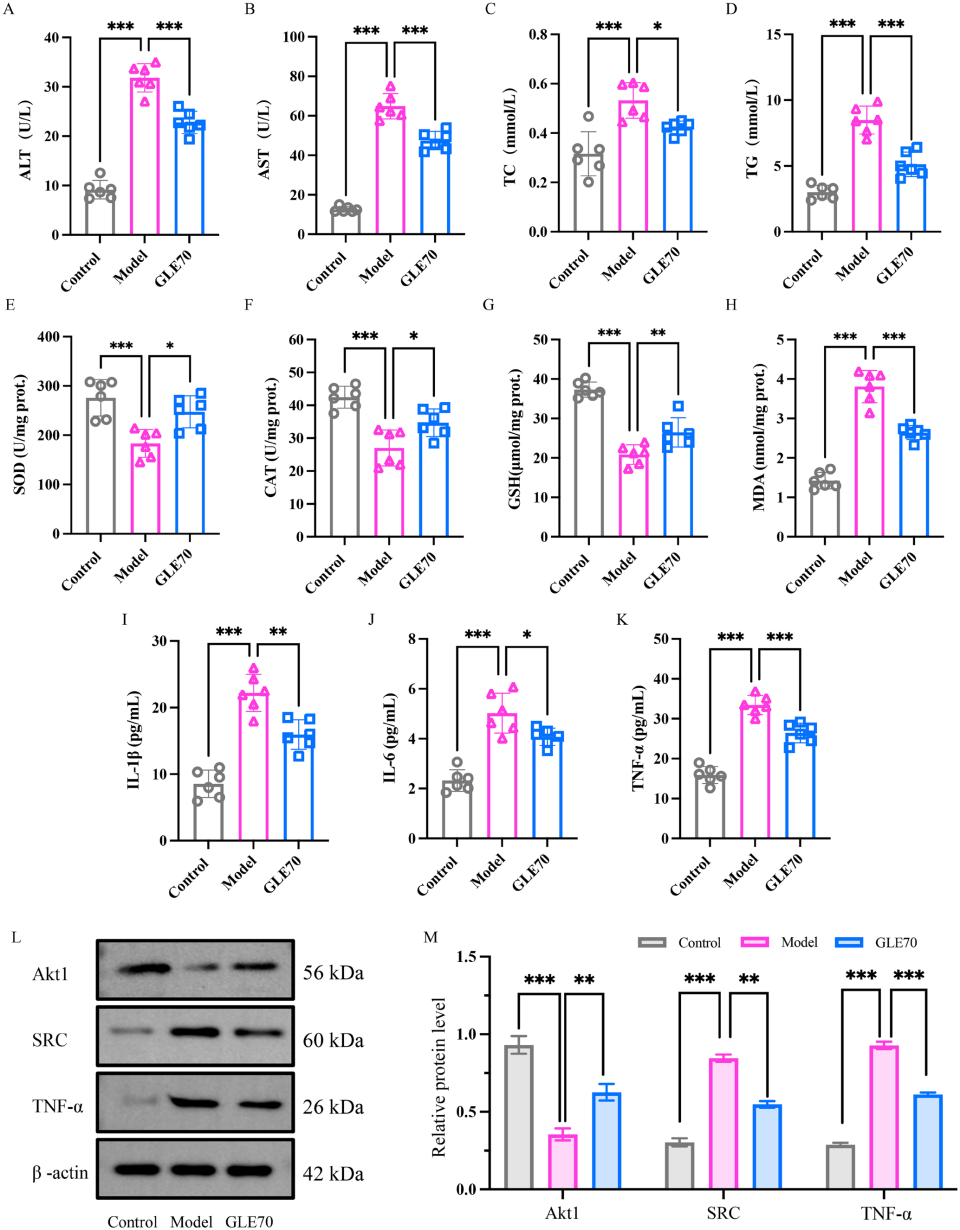
Serum levels of (A) ALT; (B) AST; (C) TC; (D) TG in each group of mice; liver levels of (E) SOD, (F) CAT, (G) GSH, (H) MDA, (I) TNF‐α, (J) IL‐1β and (K) IL‐6 in each group of mice; relative mRNA expression (L), relative protein expression (M) of AKT1, SRC, and TNF‐α in each group of mice. Note: Compared with the Model group, *p < 0.05, **p < 0.01, ***p < 0.001, the same below.

Alcohol metabolism perturbs hepatocellular membrane integrity and promotes the leakage of ALT and AST into the bloodstream (L. Qin et al. 2025). The activities of these enzymes are reliable indicators of hepatocellular injury and correlate closely with liver functional impairment. As depicted in Figure 4A,B, serum ALT and AST levels in the Model group were significantly higher than those in the Control group, increasing by approximately 3.47‐fold and 5.14‐fold, respectively (*p* < 0.05). Following GLE70 administration, ALT and AST levels were markedly reduced. GLE70 lowered serum ALT by 28.34% and AST by 26.83% (*p* < 0.05), indicating that GLE70 helps preserve hepatocyte integrity and attenuate alcohol‐induced liver injury.

Excessive alcohol intake also disrupts lipid metabolism, leading to excessive accumulation of hepatic lipids and the development of steatosis (Liu et al. 2025). As illustrated in Figure 4C,D, GLE70 intervention markedly reduced serum TC and TG levels by 20.62% and 39.56% (*p* < 0.05). These improvements suggest that GLE70 helps correct alcohol‐induced lipid dysregulation and may limit hepatic lipid accumulation. Previous studies also demonstrated that ganoderic acid A reduces alcohol‐induced elevations in ALT, AST, TG, and TC and mitigates lipid accumulation in mice (Lv et al. 2022). Taken together, these results demonstrate that GLE70 exerts a protective effect in ALD model mice by maintaining hepatocellular integrity and ameliorating lipid metabolic disturbances, thereby alleviating key pathological features associated with early‐stage ALD.

#### 3.6.2 Effects of GLE70 on Hepatic Antioxidant Defenses and Oxidative Stress in ALD Mice

Alcohol‐induced oxidative stress weakens hepatic antioxidant defenses by lowering SOD, CAT, and GSH levels, and simultaneously promotes lipid peroxidation, leading to increased MDA formation (Chumroenvidhayakul et al. 2023; Qin et al. 2024). As presented in Figure 4E–H, the Model group displayed significantly reduced hepatic SOD, CAT, and GSH levels together with elevated MDA (*p* < 0.05), which confirms the presence of marked oxidative stress. Chronic alcohol exposure is known to generate acetaldehyde and other toxic metabolites that intensify oxidative damage and enhance MDA accumulation, ultimately contributing to hepatocellular injury (Yan et al. 2024). Compared with the Model group, GLE70 treatment significantly restored hepatic antioxidant capacity (*p* < 0.05). SOD, CAT, and GSH increased by 35.08%, 28.37%, and 27.02%, respectively, while MDA decreased by 30.68%. These improvements are in line with previous findings showing that Ganoderma triterpenoids enhance antioxidant enzyme activities and mitigate alcohol‐related oxidative injury (Cao et al. 2022). Overall, the data indicate that GLE70 strengthens hepatic antioxidant defenses, reduces lipid peroxidation, and alleviates alcohol‐induced oxidative damage in ALD model mice.

#### 3.6.3 Effects of GLE70 on Hepatic Pro‐Inflammatory Cytokines in ALD Mice

The overexpression of pro‐inflammatory cytokines in hepatic tissue is a central contributor to inflammation and liver injury in ALD. TNF‐α is particularly important because it activates the NF‐κB pathway (Lai et al. 2024), promoting inflammatory signaling and hepatocyte damage. IL‐1β and IL‐6 further intensify inflammatory activity and are closely linked to lipid accumulation, hepatocellular stress, and fibrosis (Xie et al. 2024). As shown in Figure 4I–K, the Model group exhibited markedly elevated hepatic TNF‐α, IL‐1β, and IL‐6 levels compared with the Control group (*p* < 0.05), reflecting pronounced inflammatory infiltration in the liver. GLE70 administration significantly mitigated this inflammatory response. Relative to the Model group, TNF‐α, IL‐1β, and IL‐6 levels were reduced to 28.19%, 18.85%, and 20.91%, respectively (*p* < 0.05), demonstrating substantial suppression of cytokine overproduction. Similar anti‐inflammatory effects have been reported for Ganoderma triterpenoid‐enriched extracts, which markedly decreased TNF‐α, IL‐1β, and IL‐6 levels and improved alcohol‐induced hepatic injury (Zhang et al. 2024). Collectively, these results indicate that GLE70 effectively attenuates alcohol‐induced hepatic inflammation by downregulating key pro‐inflammatory cytokines and thereby alleviating liver injury.

#### 3.6.4 Effects of GLE70 on AKT1, SRC and TNF‐α Protein Expression in ALD Mice

To verify the involvement of these targets predicted by network pharmacology, Western blot analysis was performed to assess their protein expression in liver tissues. As shown in Figure 4L, the Model group exhibited a pronounced increase in SRC and TNF‐α expression accompanied by a marked reduction in AKT1, indicating substantial activation of inflammatory pathways and suppression of cytoprotective mechanisms (*p* < 0.05). In contrast, GLE70 administration significantly modulated this dysregulated expression pattern. Quantitative analysis using ImageJ (Figure 4M) demonstrated that GLE70 reduced SRC and TNF‐α protein levels to 35.14% and 34.13% of those in the Model group, respectively, while AKT1 expression increased by 51.11% (*p* < 0.05). These findings confirm that the predicted targets AKT1, SRC, and TNF are coordinately regulated at the protein level, supporting their roles in alleviating oxidative stress and inflammatory responses. The reduction in TNF‐α was accompanied by decreased levels of IL‐1β and IL‐6, further indicating its involvement in downstream inflammatory signaling in ALD. Previous studies provide additional support for this multi‐pathway regulatory action of Ganoderma triterpenoids. Zhao et al. demonstrated that GLE afforded hepatoprotection in alcohol‐exposed mice through multiple mechanisms, including the inhibition of lipid peroxidation, enhancement of antioxidant defenses, suppression of apoptosis, and modulation of immune–inflammatory responses (Zhao et al. 2019). Similarly, Cao et al. reported that triterpenoid‐enriched GLE markedly ameliorated alcohol‐induced hepatic metabolic disturbances and regulated the expression of genes involved in alcohol metabolism, lipid synthesis and breakdown, oxidative stress, and bile acid homeostasis (Cao et al. 2022). These findings collectively underscore the importance of multi‐component synergy in *G. lingzhi* extracts, wherein structurally diverse triterpenoids collectively regulate numerous biological pathways and molecular targets to confer hepatoprotection.

## 4 Conclusion

In this study, ultrasound‐assisted enzymatic extraction markedly increased the triterpenoid yield of *G. lingzhi* (16.32 ± 0.22 mg/g), and AB‐8 macroporous resin gradient ethanol elution enabled efficient enrichment of bioactive triterpenoid fractions. Functional activity screening then identified GLE70 as the fraction with the strongest hepatoprotective potential. Eight major triterpenoids were identified by HPLC–TOF–MS/MS, among which Ganoderic acids B and D were present at relatively higher levels. Integrated network pharmacology, molecular docking, and validation in an ALD mouse model suggest that GLE70 exerts coordinated antioxidant, anti‐inflammatory, and metabolic regulatory effects, possibly through AKT1 activation and suppression of TNF‐α/SRC‐related pathways. Overall, this work provides experimental evidence for the hepatoprotective effects of GLE70 and offers a practical approach for identifying bioactive fractions of *G. lingzhi*.

## Author Contributions

**Wenjia Li:** data curation, methodology. **Ping Chen:** conceptualization, funding acquisition, supervision, writing – review and editing. **Biying Zhang:** conceptualization, supervision, writing – review and editing. **Yanjun Chen:** conceptualization, data curation, formal analysis, visualization, writing – original draft. **Jiaxue Li:** data curation, formal analysis, validation. **Shiyi Ye:** validation, visualization. **Xiaobing Song:** conceptualization. **Qianli Ma:** resources.

## Funding

This work was supported by Zhongke Special Food Institute (2022220001000002).

## Supporting information


**Table S1:** Results of the orthogonal experiment on the dosage of the enzyme complex.
**Table S2:** Analysis of variance.
**Table S3:** Results of the response surface experiment.
**Table S4:** Analysis of variance results for response surface.
**Figure S1:** Influence of individual factors on the total triterpenoid yield of GLE. (A) pH for enzymatic hydrolysis, (B) Extraction temperature, (C) Extraction time, (D) Ethanol concentration.
**Figure S2:** Results of the response surface experiment, (A) pH and extraction temperature, (B) pH and extraction time, (C) pH and ethanol concentration, (D) Extraction temperature and extraction time, (E) Extraction temperature and ethanol concentration, (F) Extraction time and ethanol concentration.
**Figure S3:** Ms/ms spectrum of (A) Ganolucidic acid B; (B) Ganoderic acid B; (C) Ganoderic acid LM2; (D) Ganoderic acid D; (E) Ganoderenic acid C; (F) 9 (11)‐Dehydromanogenin; (G) 4,4,8,10,14‐Pentamethyl‐17‐(4,5,6‐trihydroxy‐6‐methylheptan‐2‐yl)‐2,5,6,7,9,15‐hexahydro‐1H‐cyclopenta [a]phenanthrene‐3,16‐dione; (H) Ganodermic acid Jb.

## Data Availability

Data sharing not applicable to this article as no datasets were generated or analyzed during the current study.
