# Supplementary material for: A Triterpenoid‐Enriched GLE70 Fraction From Ganoderma lingzhi Ameliorates Alcoholic Liver Disease via Multi‐Target Regulation
Source: Food Sci Nutr. 2026 May 14;14(5):e71893. doi: 10.1002/fsn3.71893 (PMC13176464; doi:10.1002/fsn3.71893)
Supplement: Supplementary file 1 — Table S1: Results of the orthogonal experiment on the dosage of the enzyme complex. Table S2: Analysis of variance. Table S3: Results of the response surface experiment. Table S4: Analysis of variance results for response surface. Figure S1: Influence of individual factors on the total triterpenoid yield of GLE. (A) pH for enzymatic hydrolysis, (B) Extraction temperature, (C) Extraction time, (D) Ethanol concentration. Figure S2: Results of the response surface experiment, (A) pH and extraction temperature, (B) pH and extraction time, (C) pH and ethanol concentration, (D) Extraction temperature and extraction time, (E) Extraction temperature and ethanol concentration, (F) Extraction time and ethanol concentration. Figure S3: Ms/ms spectrum of (A) Ganolucidic acid B; (B) Ganoderic acid B; (C) Ganoderic acid LM2; (D) Ganoderic acid D; (E) Ganoderenic acid C; (F) 9 (11)‐Dehydromanogenin; (G) 4,4,8,10,14‐Pentamethyl‐17‐(4,5,6‐trihydroxy‐6‐methylheptan‐2‐yl)‐2,5,6,7,9,15‐hexahydro‐1H‐cyclopenta [a]phenanthrene‐3,16‐dione; (H) Ganodermic acid Jb. [file FSN3-14-e71893-s001.docx]

**Supplementary Material**

**Table S1.** Results of the orthogonal experiment on the dosage of the enzyme complex.

| No. | A Neutral protease | B Hemicellulases | C Papain | Total triterpenes yield  (mg/g) |
| --- | --- | --- | --- | --- |
| 1 | 1.5 | 0.5 | 0.5 | 15.31±0.28 |
| 2 | 1.5 | 1.0 | 1.0 | 15.19±0.39 |
| 3 | 1.5 | 1.5 | 1.5 | 14.83±0.13 |
| 4 | 2.0 | 0.5 | 1.0 | 15.37±0.45 |
| 5 | 2.0 | 1.0 | 1.5 | 15.46±0.39 |
| 6 | 2.0 | 1.5 | 0.5 | 14.10±0.32 |
| 7 | 2.5 | 0.5 | 1.5 | 14.52±0.49 |
| 8 | 2.5 | 1.0 | 0.5 | 14.23±0.09 |
| 9 | 2.5 | 1.5 | 1.0 | 14.64±0.58 |
| K_1_ | 45.330 | 45.200 | 43.640 |  |
| K_2_ | 44.930 | 44.880 | 45.200 |  |
| K_3_ | 43.390 | 43.570 | 44.810 |  |
| k_1_ | 15.110 | 15.067 | 14.547 |  |
| k_2_ | 14.977 | 14.960 | 15.067 |  |
| k_3_ | 14.463 | 14.523 | 14.937 |  |
| R | 0.647 | 0.543 | 0.390 |  |

**Supplementary Table S2.** Analysis of variance.

| Source | Sum of squared deviations | Df | Mean Square | F Value | *p*-Value |
| --- | --- | --- | --- | --- | --- |
| A | 0.699 | 2 | 0.3495 | 1.6106 | 0.383 |
| B | 0.497 | 2 | 0.2485 | 1.1452 | 0.466 |
| C | 0.439 | 2 | 0.2195 | 1.0116 | 0.487 |
| Error | 0.434 | 2 | 0.2170 |  |  |

**Supplementary Table S3.** Results of the response surface experiment.

| No. | X_1_  Enzymatic digestion pH | X_2_  Extraction temperature（℃） | X_3_  Extraction time  （min） | X_4_  Ethanol concentration（%） | Total triterpenes yield  （mg/g） |
| --- | --- | --- | --- | --- | --- |
| 1 | -1 | 0 | 0 | -1 | 14.12±0.23 |
| 2 | -1 | 0 | 1 | 0 | 14.78±0.16 |
| 3 | -1 | 1 | 0 | 0 | 15.20±0.34 |
| 4 | -1 | -1 | 0 | 0 | 14.90±0.21 |
| 5 | -1 | 0 | -1 | 0 | 15.31±0.40 |
| 6 | -1 | 0 | 0 | 1 | 14.76±0.31 |
| 7 | 0 | -1 | 0 | -1 | 14.68±0.29 |
| 8 | 0 | 0 | 1 | -1 | 14.84±0.30 |
| 9 | 0 | 0 | -1 | -1 | 15.05±0.70 |
| 10 | 0 | 1 | 0 | -1 | 15.17±0.16 |
| 11 | 0 | -1 | 1 | 0 | 15.48±0.41 |
| 12 | 0 | -1 | -1 | 0 | 15.50±0.45 |
| 13 | 0 | 0 | 0 | 0 | 16.29±0.09 |
| 14 | 0 | 0 | 0 | 0 | 16.25±0.06 |
| 15 | 0 | 0 | 0 | 0 | 16.32±0.29 |
| 16 | 0 | 0 | 0 | 0 | 16.34±0.59 |
| 17 | 0 | 0 | 0 | 0 | 16.30±0.47 |
| 18 | 0 | 1 | 1 | 0 | 15.81±0.19 |
| 19 | 0 | 1 | -1 | 0 | 16.04±0.61 |
| 20 | 0 | -1 | 0 | 1 | 15.42±0.20 |
| 21 | 0 | 0 | 1 | 1 | 15.38±0.11 |
| 22 | 0 | 0 | -1 | 1 | 15.59±0.45 |
| 23 | 0 | 1 | 0 | 1 | 15.63±0.85 |
| 24 | 1 | 0 | 0 | -1 | 14.56±0.18 |
| 25 | 1 | -1 | 0 | 0 | 15.23±0.24 |
| 26 | 1 | 0 | 1 | 0 | 15.54±0.47 |
| 27 | 1 | 0 | -1 | 0 | 15.26±0.09 |
| 28 | 1 | 1 | 0 | 0 | 15.51±0.44 |
| 29 | 1 | 0 | 0 | 1 | 14.95±0.39 |

**Supplementary Table S4.** Analysis of variance results for response surface.

| Source | Sum of Squares | Df | Mean Square | F Value | *p*-Value |
| --- | --- | --- | --- | --- | --- |
| Model | 9.41 | 14 | 0.6722 | 194.95 | <0.0001** |
| A | 0.3267 | 1 | 0.3267 | 94.74 | <0.0001** |
| B | 0.3852 | 1 | 0.3852 | 111.71 | <0.0001** |
| C | 0.0705 | 1 | 0.0705 | 20.46 | 0.0005** |
| D | 0.9130 | 1 | 0.9130 | 264.78 | <0.0001** |
| AB | 1.000E-004 | 1 | 1.000E-004 | 0.0290 | 0.8672 |
| AC | 0.1640 | 1 | 0.1640 | 47.57 | <0.0001** |
| AD | 0.0156 | 1 | 0.0156 | 4.53 | 0.0515 |
| BC | 0.0110 | 1 | 0.0110 | 3.20 | 0.0954 |
| BD | 0.0196 | 1 | 0.0196 | 5.68 | 0.0318* |
| CD | 1.776E-15 | 1 | 1.776E-15 | 5.152E-13 | 1.0000 |
| A^2^ | 4.48 | 1 | 4.48 | 1299.81 | <0.0001** |
| B^2^ | 0.4905 | 1 | 0.4905 | 142.26 | <0.0001** |
| C^2^ | 0.4861 | 1 | 0.4861 | 140.97 | <0.0001** |
| D^2^ | 4.44 | 1 | 4.44 | 1288.11 | <0.0001** |
| Residual | 0.0483 | 14 | 0.0034 |  |  |
| Lack of fit | 0.0437 | 10 | 0.0044 | 3.80 | 0.1051 |
| Pure error | 0.0046 | 4 | 0.0012 |  |  |
| Cor total | 9.46 | 28 |  |  |  |

**Supplementary Fig. S1.** Influence of individual factors on the total triterpenoid yield of GLE. (A) pH for enzymatic hydrolysis, (B) Extraction temperature, (C) Extraction time, (D) Ethanol concentration.

**Supplementary Fig. S2.** Results of the response surface experiment, (A) pH and extraction temperature, (B) pH and extraction time, (C) pH and ethanol concentration, (D) Extraction temperature and extraction time, (E) Extraction temperature and ethanol concentration, (F) Extraction time and ethanol concentration.

**Supplementary Fig. S3.** Ms/ms spectrum of (A) Ganolucidic acid B; (B) Ganoderic acid B; (C) Ganoderic acid LM2; (D) Ganoderic acid D; (E) Ganoderenic acid C; (F) 9(11)-Dehydromanogenin; (G) 4,4,8,10,14-Pentamethyl-17-(4,5,6-trihydroxy-6-methylheptan-2-yl)-2,5,6,7,9,15-hexahydro-1H-cyclopenta[a]phenanthrene-3,16-dione; (H) Ganodermic acid Jb.
